# Dr. Alonzo B. Richardson

**Published:** 1903-08

**Authors:** 


Dr. Alonzo B. Richardson, medical superintendent of the Government Hospital for the insane at Washington, D. C., died in that city June 27, 1903, from apoplexy, aged 50 years. Dr. Richardson was a native of Ohio and one of the most prominent alienists in the United States. He was president of the American Medico-Psychological Association at the time of his death.

